# Implementing Health Impact Assessment at National Level: An Experience in Iran

**Published:** 2018-02

**Authors:** Behzad DAMARI, Abbas VOSOOGH-MOGHADDAM, Sahand RIAZI-ISFAHANI

**Affiliations:** 1. Dept. of Social Determinants of Health, National Institute of Health Research, Tehran University of Medical Sciences, Tehran, Iran; 2. Ministry of Health and Medical Education, Tehran, Iran

**Keywords:** Health impact assessment (HIA), Implementation, Developing countries, Iran

## Abstract

**Background:**

According to the general health policies issued in 2014, Health Impact Assessment (HIA) or Health Annex should be implemented in Iran. The present study provided a model for executing HIA in the Iranian context as a developing country.

**Methods:**

This is a system design study with the qualitative approach. The data on the system components were gathered via reviews of the literature, in-depth interviews and focused group discussions (FGDs) with experts. The information were contently analyzed in order to draft the model and a consensus was reached on by the steering committee.

**Results:**

Fifteen in-depth interviews and six FGD meeting were conducted. The equity-based approach in assessing the health impacts of policies, programs and projects were chosen as the most practical tool. Experts believe that for the next five years, HIA should be used just for the “national projects” so that the ministries and national agencies could be empowered. Components of the model including structure, procedures, and standards, management style, mission and resources were prepared. The national regulations and protocols were sent to the SCHFS Secretariat for final revision and the council approval.

**Conclusion:**

The hasty implementation of HIA will face serious resistances as the health-oriented attitude and behavior in both government and non-governmental sectors will gradually form. Also, the overlapping of the contents of HIA with other tools such as Environmental, Cultural and Social Impact Assessments, currently used by other sectors, causes difficulties in implementing the HIA by the Ministry of Health and Medical Education.

## Introduction

Enjoyment of highest level of health is emphasized by WHO as one of the basic human rights (1). This right bounds the governments to take steps in order to provide the opportunities to achieve health for all (2). The impact of health on economic and social issues has made it the cornerstone of sustainable development (3, 4). In fact, public health is the ultimate goal for social, economic, and educational systems activities in each society (3). By adopting health in all policies strategy, different sectors can smartly focus on the impact of their actions on health of each demographic group. The main goal of this strategy is to improve public health with an extensive attention to the social determinants of health (5).

Health Impact Assessment (HIA) is a combination of procedures and methods used as a tool to judge the impact of a given policy, program or project on the health of the human population (6). The HIA is meant to result in evidence-based recommendations for informing decision-makers (7, 8). These recommendations are aimed to maximize positive impacts and minimizing negative impacts of the policy, program or project on health.

There are different approaches to implementing HIA. In Finland, the combination of health and social impact assessment is called Human Impact Assessment, which is a voluntary action (5). In countries such as the USA, Denmark, Spain, and Thailand, HIA is conducted independently, while in Canada, Italy, and Sweden it is integrated with EIA; In UK Scotland and Wales, various types of HIA are used known as Equality Impact Assessments (EqIA) (9–11). Moreover, an integrated model of environmental, social, and health impact assessment (ESHIA) is used in some countries for important projects such as oil and gas (12).

HIA can be performed in three stages: before, during or after implementation of the policy, program or project. Although there is no agreement on the best stage, performing HIA before the implementation provides more opportunities (13).

The International Association for Impact Assessment (IAIA) proposed five principles and values for HIA (14). Respecting the public rights for participation in the decision-making process probably is the most important value; that is the public or their representatives especially those intentionally or unintentionally influenced should participate in the decision making process. In the recent decade, as the notion of “equity in health” became more popular, the equity-based HIA was introduced. This model pays more attention to the impacts of policy, program or project on the vulnerable health groups and encourages policymakers to consider different groups in implementing interventions (15).

As the HIA strategy has been repeated in the 5^th^ 5 yr development plan act, the general health policies and even in the general policies of the coming 6^th^ 5 yr development plan (2017–2021), the aim of this study was to provide an executive model for implementing “Health Annex” effectively at national level in Iran.

## Methods

This survey is a model design with the qualitative approach. The required data was gathered by reviewing literature and published experiences, in-depth interviews with informed people, getting feedbacks from representatives of SCHFS and focused group discussions with experts. Experiences of EU, Canada, US, and Australia are studied and summarized. In addition, the lessons learned from implementation of EIA in Iran were investigated. The search keywords were Health Impact Assessment, HIA, Health Annex, equity health impact assessment, social impact assessment, organization accountability, Environment Impact Assessment.

**Fig. 1:**
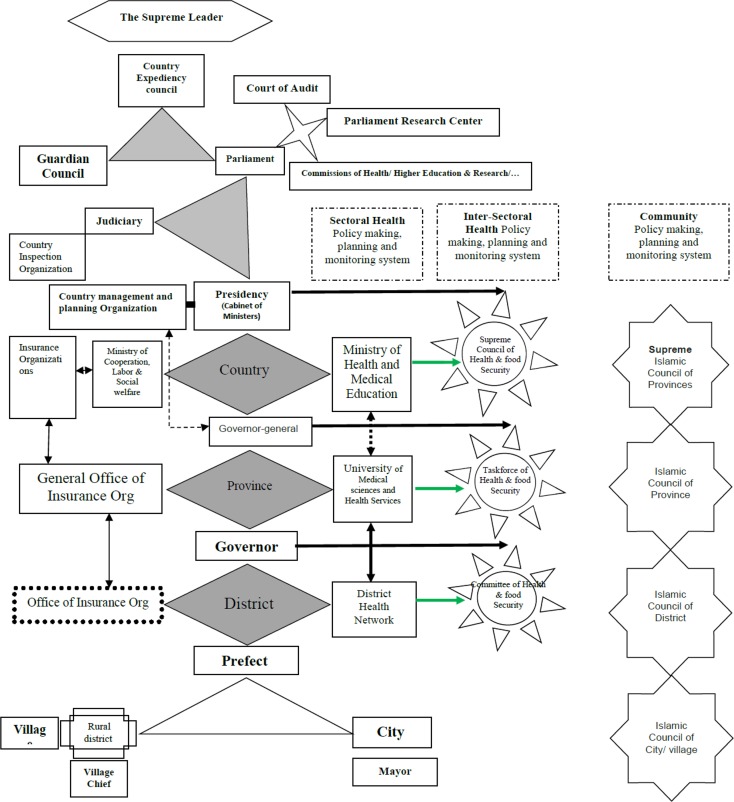
Organizations, and governmental and community councils involved in health policymaking, planning and monitoring at national, provincial and districts in Iran

**Fig. 2:**
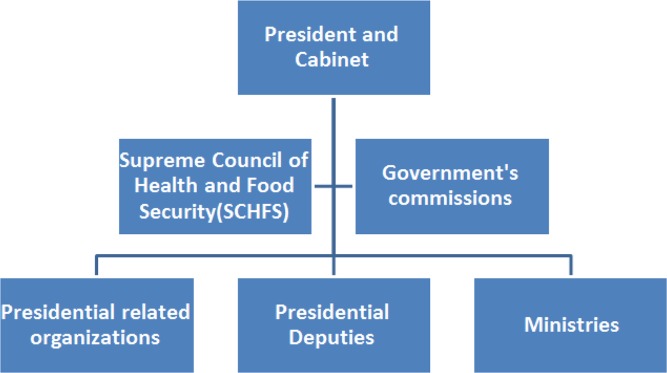
Position of Supreme Council of Health and Food Security (SCHFS) in Iran state structure

Fig. 1 shows the organizations, and governmental and community councils involved in health policymaking, planning and monitoring in Iran. After a stakeholder analysis, based on the power/influence and interest of the individuals, major members of permanent commission of the SCHFS were selected as the Steering Committee. The position of SCHFS in Iran state structure is shown in Fig. 2. The task of this committee was the coordination for collecting data and ideas from the organizations for implementing HIA, approving the design principles and supporting the HIA charter.

The opinions of the members of the permanent commission of the SCHFS on the necessity and the way of establishment were taken using an interview (Box 1).

The interviews were conducted from September 2014 to February 2015. All principles of structured interviews including ensuring representation of stakeholders, prior notice to the interviewees, justification of interviewees and giving evidence-based information on the state of the country, the interview guide, selecting an interviewer familiar with the research content, consent of interviewee for recording the interview, transcribing the voice of interviewee immediately after the interview was followed. The interview data were analyzed and classified following determination of main subjects.

Besides, six experts were selected for participating in focused group discussions (FGDs) based on following criteria:

1. Administrative – academic pioneer in the national health system (2)
2. Being informed of (senior management or expert) the President's Office Deputy of Strategic Planning and Control (2)
3. Public health specialist introduced by Iranian Association of Social Medicine (2)

Overall, six FGD sessions were held from February to June 2015. In each session, the questions were raised by a facilitator, and by assigning a member as the session manager, ideas and opinions were collected. The discussions were recorded with the group's consent and transcribed immediately after the sessions and a partial draft of the model was prepared. At the beginning of the next session, this draft was discussed. In the first session, a consensus was reached on the definitions on the formulation of HIA (Box 2) and the guidelines for designing HIA establishment model. In the following sessions, the components of the model including structure, processes, standards and management style, mission and resources were prepared and the protocols were sent to SCHFS Secretariat for final revision and the council approval.

## Results

Table 1 shows the amount of agreement of the representatives of organizations and ministries presented in SCHFS on the necessity for implementation of HIA in the country. The majority of the members (14 out of 15) ‘highly agreed’ or ‘agreed’. The representative of the Ministry of Interior expressed: “I do not agree with the term “Health Annex”, but I consider presence of mechanisms for monitoring and assessing programs and projects”. Other members also suggested some modifications for the effective implementation of HIA including “measurable parameters should be used and international standards should be taken into account” and “A common concept should be agreed upon by all stakeholders”.

**Table 1:** Viewpoints of the representatives of members of different ministries and organizations regarding necessity for establishment of health impact assessment

	***Organizations***	***Highly Agree***	***Agree***	***No Comments***	***Disagree***	***Highly Disagree***
1	Ministry of Trade		√			
2	Ministry of Justice	√				
3	Ministry of Education		√			
4	Ministry of Energy(Water Authority)		√			
5	Ministry of Economic Affairs and Finance		√			
6	Ministry of Energy	√				
7	Islamic Republic of Iran Broadcasting		√			
8	Imam Khomeini Relief Foundation	√				
9	Ministry of Cooperatives, Labor, and Social Welfare		√			
10	Ministry of Industries and Mines	√				
11	Ministry of Agriculture		√			
12	Physical Training Org	√				
13	Iran's Management and Planning Organization		√			
14	Iranian Department of Environment	√				
15	Ministry of Interior				√	
	Summary	6	8		1	

There were different opinions on how to implement HIA. The compulsory approach was the most agreed. Overall, the respondents believed that: The concept of HIA has not yet become popular. “First, HIA should become more popular and then it can be implemented in a more suitable time period”. “Hastiness should be avoided and the processes should be specified so that HIA does not meet the same fate as EIA”. “HIA is an intersectoral matter and it should be binding. The SCHFS Secretariat should be the responsible authority and prepare the HIA through SCHFS in consultation with respective bodies”. “Administrative Regulations should be developed for HIA and supervised by the Ministry of Health. There also should be a senior advisor in every organization”. “HIA reports should be prepared through agencies approved by SCHFS. HIA and the way of its implementation should be monitored and followed up in developmental projects of administrative bodies, and the agencies should announce the results”. “Development of HIA is a new and novel issue, for its implementation, either existing human resources should be trained adequately, or new and organized forces should be recruited”.

“Our organization is implementing the bylaw of EIA since 1994, but there are some problems,” Mentioned the representative of IEPA”. “Supervision over implementation of the projects is not done based on the Prepared EIA reports. In other words, the recommendations in the EIA are not monitored, and the report may just be placed on the library shelves. Moreover, the EIA report should be operational. Instead of addressing theories, it should provide practical solutions so that investors and policymakers do not become disappointed and totally stop the project. Above all, after about two decades, we have not yet assessed the EIA policy and not reviewed our work”.

Considering the common models (16), four main proposed processes of HIA were: screening, defining service descriptions, Report Formulation and Audit, and application.

### Stage 1: Screening

The projects, programs, and policies that require HIA are listed annually by the Health and Treatment Office of Management and Planning Organization of Iran (MPO) and sent to the National Committee of Health Annex. After the committee’s approval, the list would be sent to the SCHFS for the execution. This stage is completed with announcement of the necessary HIA list. Management of this stage is under the jurisdiction of SCHFS.

### Stage 2: defining service descriptions

The agency or ministry responsible for the policy, program or project is bound to prepare the HIA report within 6 months through the reliable consultant. This consultant is bound to respect the framework and methods mentioned in the national guideline for Health Annex. This guideline is notified by the SCHFS Secretariat to all organizations. This stage is completed by sending the formulated report to SCHFS Secretariat.

### Stage 3: Report Formulation and Audit

The HIA report is sent to National Committee on Health Annex. This Committee consists of at least three executive members (including health deputy of minister as the head, chairman of SCHFS Secretariat, and representative of Management and Planning Organization of Iran (MPO) as supervisor) and five academic members in different fields related to physical, mental, social, spiritual, and environmental health. Two scenarios are imaginable for each HIA report: 1) Acceptance of the report and notification to the respected organization for implementation 2) Conditional acceptance of report (it is returned to the executive branch and the consultant for completion).

### Stage 4: Application

A senior manager in the organization, which establishes the policy, program or plan, is the first supervisor over implementation of HIA report with the consultation of the health liaison of executive branch. The health liaison is bound to provide an annual report of the application of recommendations and deliver it to SCHFS Secretariat following approval of the respective managers. This stage is completed after the investigation of SCHFS regarding annual report as well as quality and quantity of the recommendations application.

Based on the experts’ opinions and review of literature, the consultant should complete Table 2 through collection and analysis of quantitative data. HIA report includes three sections: introduction, impacts, and recommendations.

**Table 2:** Proposed format for submitting health impact assessment information in Iran agreed by the stakeholders

***Title of policy, program or project (a 100-word description)***			
Target population:			
Stakeholders:			
Domain	Variable	What is the evidence? (type of evidence: quantitative, qualitative, national, international, experts and the reference should mention)	Description of the impacts on health and the mechanism
**Negative impacts:** Does the policy, program or project under study have major negative impacts on equality in each component?	Age		
	Gender		
	Disability		
	Race/ethnicity		
	Religion and beliefs		
	Sexual orientation		
	Socio-economic class (income)		
	Geography (rural, suburban, ...)		
**Positive impacts:** Does the policy, program or project under study have major positive impacts on reducing inequalities?	Improving equal opportunities		
	Reducing discrimination		
	Reduced harassments		
	Promoting good social relations		
	Promoting positive attitudes towards disabled people		
	Encouraging participation by disabled people		
	Consideration of better treatments for people with disabilities		
	Promotion and protection of human rights		
What should be done? (types of interventions)	1. Structural (addressing the root causes of health inequalities)		
	2. connector ways (mediator)		
	3. Health and Disability Services		
	4. Minimizing the impact		
	Summarized Interventions: national regional and local level / individual and population-based approaches		

The structure of HIA establishment in the country is clarified in the legal acts of the Parliament and the Cabinet. Article 32 of Fifth Development Plan, Act of Cabinet on job description of SCHFS and the Article 6, which permit formation of specialized taskforces by suggestion of the SCHFS, all predict components of the structure.

There are several theories for effective implementation of policies and strategies. It was agreed that the “Evaluation framework” (17) is the more appropriate method in order to implement HIA in a multi-stage pattern.

## Discussion

Our findings showed that there is a positive attitude in members of the permanent commission of SCHFS toward HIA establishment. Although many different tools and models exist in the world for assessment of health impacts of policies, programs, and projects, it was necessary to redesign a local model suited for the country context. Paying special attention to the national policymaking and planning system was one of the guiding criteria in designing of this model. No new structure was proposed for establishment of this strategy, and available assets in the national health policymaking system were used. Motivation of all stakeholders was also considered. The processes of accreditation of consultants, evaluation of reports mean using various experts in National Committee of Health Annex. The Interaction between the executive branch and the contractor with the consultant in preparing HIA report, notification of the one-page recommendations of National Committee of Health Annex, and using health liaisons in the respective organizations will considerably guarantee the application of the results.

Although the experiences of EIA establishment were used in the designing of this model, one of the major challenges of HIA establishment in the country is the overlap between content of HIA and other current approved tools including EIA (Iranian Department of Environment), Cultural Impact Assessment (the responsible organizations is the Supreme Council of the Cultural Revolution) as well as Social Impact Assessment (municipalities). The cabinet formulates and announces new regulations for integration of these Annexes respecting their logical share and standards.

Although HIA can be prepared for policies, programs or projects (18), but using HIA tool for “projects” is more practical than policies or programs, because the framework of public policies and programs is often variable, biased and sometimes unwritten, and there is no formulated identity, thus the assessment is difficult; While there is a defined and operational system for projects announced by the President’s Office Deputy of Strategic Planning and Control.

The scope of the model was defined to the administrative bodies outside the Ministry of Health and Medical Education, which brings about two challenges: a) It does not include the policies, programs or projects of the Ministry of Health itself. b) It does not include legislative and judicial powers and the organizations under their supervision. The opportunity to collect the ideas of organizations on the formulated model was not provided in the current study and complementary studies are required.

Another challenge for establishment of the designed system of HIA is that if the government does not want to assess the policy, program or project, then who can force it. This is a global issue as planning decisions are usually made by the bodies outside the health sector (18). This challenge is resolved by formulation and approval of a public law, which guarantees participation of supervisory bodies and a better intersectoral collaboration.

Empowerment of human resource is considered as a basic prerequisite for HIA establishment (19, 20). In this regard, Ministry of Health and Medical Education should include the required knowledge in curriculums related to public health such as social medicine, health policy making, and epidemiology, and develop interdisciplinary research.

The following steps should be taken for application of results of this study:

- Approval of the bylaw resulting from this study in SCHFS
- Formation of National Committee of HIA and assignment of the members by the secretary vice president of Health and Food Security Supreme Council
- Formulation of permission issuance bylaw for consultants, and requirements which should be complied by the consultant; formulation and announcement of technical guideline for HIA formulation
- appointing the experts of SCHFS Secretariat and health liaisons for the organizations
- Training experts and health liaisons for the respective organizations
- Pilot implementation of the process for five volunteer organizations and the evaluating the process and its components and changing bylaw if necessary
- the experience of Thailand in implementing HIA showed that an important obstacle to HIA development is incomplete and dispersed information on health status and environment indicators, (21) so it is required that reliable sources of data were secured.

Sometimes health is not the primary force driving the decision to implement a policy, program or project, thus HIA allows the decision makers to consider the health impacts of other factors (18).

Binding projects, programs, and policies to perform HIA reports in Iran is often faced with difficulty and resistance (22). In other words, the health-oriented behavior in governmental and non-governmental (private, public, charity, and cooperative) sector needs some cultural modification and establishment of the belief that HIA improves the projects rather than being an obstacle for their implementation.

## Conclusion

This study showed that there are two challenges in implementing the HIA in Iran:

1. The health-oriented attitude and behavior is not completely formed in both government and non-governmental sectors, therefore the implementation of HIA will face resistances.
2. There are many overlaps between the contents of HIA and other tools such as Environmental, Cultural and Social Impact Assessments, currently used by other sectors.

## Ethical considerations

Ethical issues (Including plagiarism, informed consent, misconduct, data fabrication and/or falsification, double publication and/or submission, redundancy, etc.) have been completely observed by the authors.

## Appendices

Box 1: The interview questions:

1. How much do you agree on the necessity of establishment of Health Impact assessment (HIA) in general?
2. How much do you agree on the establishment of HIA in a self-monitoring manner: through council of deputies of ministries / organizations with the consultation of public health experts?
3. How much do you agree on the establishment of HIA in an advisory manner: through the President’s Deputy of Strategic Planning and Control Office and Supreme Council of Health and Food Security (SCHFS)?
4. How much do you agree on the establishment of HIA in a compulsory manner: through SCHFS?

Box 2: The agreed definitions on the formulation of HIA

1. Health Impact Assessment (HIA) Protocol: The requirements for process and product that must be met by the consultant in formulating HIA.
2. Executive Body: The organization or ministry that is the overseer of the policy, program or project. The executive body has role of the employer.
3. Private Executer: The contractor or investor in the establishment of a policy, program or project
4. Consultant: A real or legal person licensed to develop the HIA.
5. Health liaison: An expert person in an organization or ministry, which is responsible for advocacy and facilitation of health inter-sectorial coordination and is appointed by shared orders of minster of Health and collaborating organization or ministry head.
6. Implementation Report: the middle or final report on the results of the approved recommendations (as contained in the HIA report) employed by the Executive body.
7. National Committee of HIA: A specialized committee for overseeing the main processes for formulation and application of HIA in the country.
8. Supervisor: Supervisor company in the development projects
